# Outcome assessment of a triangular clinic as a harm reduction intervention in Rajaee-Shahr Prison, Iran

**DOI:** 10.1186/1477-7517-10-41

**Published:** 2013-12-26

**Authors:** Rahim Taghizadeh Asl, Babak Eshrati, Colleen Anne Dell, Kelli Taylor, Parviz Afshar, Mohammad Kamali, Ali Mirzazadeh

**Affiliations:** 1 World Health Organization, Tehran, Iran; 2 Arak University of Medical Science, Arak, Iran; 3 Department of Sociology & School of Public Health, University of Saskatchewan, Saskatoon, Saskatchewan, Canada; 4 Faculty of Medicine/Medical School, University of Calgary, Calgary, Alberta, Canada; 5 Ministry of Welfare, Tehran, Iran; 6 Iran University of Medical Science, Tehran, Iran; 7 Institute for Health Policy Studies, School of Medicine, University of California, San Francisco, USA

**Keywords:** Triangular clinic, Injection drug use, HIV/AIDS, Health belief model

## Abstract

**Background:**

Transmission of the human immunodeficiency virus (HIV) among incarcerated injection drug users (IDU) is a health epidemic in the Islamic Republic of Iran. Triangular clinics (TCs) were established in prisons as a harm reduction measure to decrease the risk of HIV transmission and other blood-borne infections. The objective of this study was to assess the immediate outcomes of one TC among male IDUs in Iran’s Rajaee-Shahr prison.

**Methods:**

This study was conducted in two stages between 2003 and 2005. In the preparatory stage, focus group data was collected to update the prison’s TC education and medical interventions and construct the self-report questionnaire. In stage two, 150 male IDUs were recruited in a closed cohort study design to assess the immediate outcome of the TC. Participants were measured at baseline and followed up to six months to measure their drug use, attitude toward and knowledge of high risk behaviours, serological conversion for HIV, HBV and HCV, and engagement in risky behaviors. The TC outcomes were determined through random urine analysis testing, a self-administered questionnaire and behaviour report cards, and viral infection testing.

**Results:**

The findings of the urine analyses indicated a minimal yet consistent decrease in drug use over the six months. The pre and post- self-administered questionnaire data relayed a modest change in IDU risky behaviours associated with sexual practices; this was greater in comparison to the knowledge and attitude measures. It was determined that age may have a detrimental effect as may viral infections (HIV and HBV) on knowledge, attitude and behavior change. Both education and employment may have a protective effect. Data collected from the self-report behaviour cards similarly showed a modest reduction in high risk practices. At the six month follow-up, only one case became HIV positive, 9 HCV and 17 HBV.

**Conclusions:**

Considering that HIV is concentrated among Iranian prisoners who inject drugs at a high level, the results of this study indicate that TCs are a possible effective intervention. However, many prisoners continued with risky behaviors even if they were participating in harm reduction measures, such as methadone maintenance therapy.

## Background

To address the IDU and HIV epidemic we need “to break the silence and change attitudes of prisoners and policy makers and [have] collaboration all around”.

-Dr. Afshar, Director General of Health Services for the Iranian Prison Organization at the 2004 International AIDS Conference.

With a population of over 75 million people in Iran, the number of individuals abusing drugs is estimated to be over one million, with approximately 200,000 to 300,000 injection drug users (IDU) [1,2]. At present, the human immunodeficiency virus (HIV) is highly concentrated among IDUs [3]. Over the past several years there has been an increase in HIV cases originating from sexual transmission in the general population [3], however, the large majority of new cases (65-79%) remain among IDU’s sharing needles [3-5]. With a growing proportion of young people making up the Iranian population and an increase in the availability of drugs, including a mounting relationship between amphetamine-type stimulant use and high risk sexual practices, the country is vulnerable to an increase in the number of new drug users, including those engaged in injecting and other high risk behaviours [5]. In turn, there is potential for a dramatic increase in the prevalence of HIV, as well as the hepatitis B virus (HBV) and hepatitis C virus (HCV) [6]. The most recent (2012) United Nations AIDS progress report by the Islamic Republic of Iran acknowledged that if the current HIV epidemic among IDUs is left unattended, it has the potential to turn into a generalized population epidemic [3].

Acknowledging the direct linkage between IDU and the spread of HIV, Iran committed to attempts to control the spread of the disease at the 2003, 2008 and 2011 United Nations General Assembly Special Sessions on HIV/AIDS. The latest data relay that “[m]easures taken over the past ten years have successfully slowed the progression of the epidemic among injection drug users”, but the HIV rate among IDUs remains at around 15% and varies across the country [3]. A surveillance survey in 2010 in Iran further revealed that “among those who had injected drugs over the last month, 36.9% had used a non-sterile needle, and 12.6% had practiced shared injection” [7]. Controlling the spread of HIV within Iranian penitentiaries is a particular concern with their concentration of IDUs as well as high risk behaviours. Foremost, the majority of incarcerated IDUs, some HIV positive, will reintegrate back into their communities, and this is a particular concern for the spread of HIV and other transmitted diseases [8].

A 2006 study by Zamani et al. reported that 94% of IDUs in Iran have been incarcerated at least once in their lifetime. Among them, 28% reported using injection drugs at least once within the prison; of these, 82% reported using a shared injection device, with 36% of these individuals being HIV-positive [9]. A 2007 study by the same authors found that in their site-specific study of Karaj Central prison [10] in Iran, 42% of prisoners reported to use intravenous drugs before their incarceration. A more recent 2013 study, reporting on 2009 data from a sample of 27 Iranian prisons, concluded that the prevalence of HIV among the prisoner population is 2.1%, and 8.1% among those who have a history of IDU [3]. They also found that only 24% of prisoners reported condom use during their last vaginal/anal sexual encounter in prison, 12.9% reported being tattooed while incarcerated, and only one in five had sufficient knowledge of the routes of HIV transmission and preventative measures [8]. Such high risk behaviours among prisoners both before and during their incarceration have turned Iranian prisons into highly prospective environments for acquiring HIV.

In response, triangular clinics (TCs) surfaced in Iranian prisons at the turn of the 21st century [11] as part of a coordinated, country-wide approach to the dual epidemic of HIV and IDU [12]. Iran has a long-standing commitment to supply reduction efforts, that is, largely enforcement efforts that aim to disrupt the manufacturing and distribution of drugs, with a history of severe punishment for drug dealing. This coincides with the correctional system’s harsh and overcrowded prison conditions, detainment of political dissidents, and carrying out of prisoner executions. However, with a high rate of IDU and an emerging HIV health epidemic, coupled with lessons learned from other countries, demand reduction efforts that focus on treatment and prevention were introduced to reduce individuals’ needs for drugs. Harm reduction clinics initiated in the community; these are drop-in centres in drug-ridden neighborhoods where former drug abusers assist with offering “needles, methadone, treatment for sexually transmitted diseases, AIDS tests and other medical care to their peers” [13]. The clinics transitioned into triangular clinics in the prison environment and similarly offer harm reduction measures. Both clinic types were embraced as both pragmatic and progressive in consideration of the country’s general conservative social climate [11,14]. In fact, Iran’s concept of the TC is largely unique across the globe [15]. In Canada and the United states, for example, the prison systems do not offer needle exchange even though IDU in these systems too is a concern; it is frequently a part of the fabric of prison life [16].

In line with Iran’s promotion of a participatory and proactive approach to control the HIV epidemic [3], TCs acknowledge the mutual role of the prisoner, the staff and the prison environment. Triangular Clinics aim to: (1) improve information to affected groups regarding HIV/AIDS/drug abuse; (2) improve information to staff regarding HIV/AIDS/drug abuse, (3) provide access to facilities regarding harm reduction, and (4) improve the quality of life and empowerment of affected citizens [15]. These aims support the Health Belief Model (HBM) and its focus on informing beliefs (knowledge) and attitudes to eventually change behaviour, and specifically within the HV/AIDS field. “Within a harm reduction context, the HBM provides a systematic framework for examining the reasoning behind an individual’s choice to decrease, maintain or increase their high risk behaviour” [17]. It is well established in the prevention and treatment literature that handing out clean needles in isolation from other support services, for example, is inadequate for sustainable behaviour change [18].

The TCs provide a variety of harm reduction services including counseling, education, referral services, and treatment such as methadone maintenance therapy (MMT) [19]. For example, MMT is currently available to opioid-dependent prisoners in over half of the 230 prisons and correctional settings in Iran [10]. With the introduction of TCs and MMT, the total number of prisoners taking part in methadone therapy increased from 100 in 2002 to more than 25,000 in 2009 [1,20]. Since their introduction they have also expanded to address STIs in addition to HIV/AIDS. However, still, relatively little research has been undertaken to assess the outcome of TC’s on behavior change. The aim of this study is to assess the immediate outcome of a TC on behavior change among injection drug users incarcerated in Rajaee-Shahr prison in Iran. Drawing on available data, the outcome indicators include (1) drug use, (2) change in attitude toward and belief about (knowledge) high risk behaviours, (3) serological conversion (HIV, HCV and HBV), and (4) engagement in risky behaviors (e.g., syringe exchange, condom use).

## Methods

### Setting and sample

In order to assess the immediate outcome of the TC as a health intervention in an Iranian prison, this study funded by the World Health Organization was undertaken in Rajaee-Shahr prison between 2003 and 2005. The lapse in time between the data collection and publication of this paper in part reflects the detailed process for data release in the country. Ethics for the study was granted from the World Health Organization, Special Programme for Research and Training in Tropical Diseases, and the Iran Prisons Organization ethics committee. Rajaee-Shahr prison is located 70 km northwest of Tehran (Iran’s capital) in the city of Karaj, has an inmate population of approximately 3,200 individuals, and is one of the most crowed and harsh prison environments in the country [12,20].

The study sample was a closed cohort of 150 incarcerated male injection drug users; the first 150 prisoners who voluntarily registered in the study were included. Given the conservative environment of the Iranian correctional facility, this was deemed the best method to solicit participation and is similar to how other topic sensitive studies have been carried out in the prison system [8]. In order to be eligible for selection, individuals must: (1) have been imprisoned for more than four months, (2) not have been diagnosed with a psychiatric disorder, (3) not have previously accessed the TC or other harm reduction services in the prison, and (4) be an injection drug user and willing to take part in MMT. Verbal informed consent was acquired from all study participants. For individuals with low literacy, a designated and trained staff member was available to confidentially assist with the prisoners’ participation in the study.

### Data collection

#### Stage one

The study was conducted in two stages: (1) the qualitative preparatory stage, and (2) the quantitative outcome testing stage. The first stage is addressed in detail in this paper and sets the necessary context for understanding the operation of a TC. In this preparatory stage, three focus groups were held. The groups were comprised of eight to ten men imprisoned in Rajaee-Shahr prison who were involved in high risk drug and sexual behaviors (e.g., needle sharing); all focus groups were held in Farsi language and lasted approximately two hours. The aim of this initial step in the study was to gather baseline understanding about the target group’s knowledge, attitude, and practices related to IDU, HIV/AIDS, HBV, HCV, unsafe sexual practices, and other risky behaviors. These three foci reflect the Health Belief Model, which focuses on individuals’ attitudes and beliefs in an attempt to explain and predict health behaviors, in particular sexual and other high risk taking to prevent the transmission of HIV [19,21,22]. This data was used to review and revise the existing TC educational and medical interventions in Rajaee-Shahr prison as well as to construct the questionnaire for the second step of the study – measuring immediate outcomes.

#### Stage two

The second stage of the study collected data from all participants at baseline and six months following their first attendance at the TC. The questionnaire was designed to address the key components of the Health Belief Model: (1) perceived risk (measuring knowledge and attitude), (2) perceived severity (measuring knowledge and attitude), (3) perceived benefits (measuring attitude), (4) perceived barriers (measuring attitude), and (4) cues to action (measuring behaviors). The questionnaire included 75 questions, measuring knowledge (39 questions), attitude (26 questions), and practices (10 questions). The content validity of the questionnaire was assessed through six expert reviews in the areas of health education, infectious disease, and psychology. The self-administered questionnaire was then tested for reliability among 40 randomly selected prisoners representative of the study target group imprisoned at Rajee-Shahr. With 34 responses, internal consistency of the questionnaire was measured with Cronbach’s Alpha test, and the outcome was greater than 74%.

In addition to the questionnaire, at pre-test each participant completed a personal history survey, a physical examination, testing for HIV, HBV and HCV viral infection, STI testing, and urine analysis for drug residue. From this, physicians determined the medical needs of each participant and suggested various treatment options, including detoxification, STI counseling, and MMT. A health and education counseling session was held with each prisoner.

All 150 participants started MMT in groups of 5 to 10 (referred to as the attack phase). Throughout this phase, participants were closely monitored for several weeks in an isolated ward of the prison to minimize any unanticipated effects of withdrawal and risks due to other drug use. The prescribed methadone dosage for each participant was determined by a physician and accounted for the participant’s health status, drug use history, and other personal indexes. The daily dosage of methadone was between 80–125 mg. Following the attack phase, participants were relocated to the common ward of the prison where they received a maintenance dose of methadone (referred to as the loading phase). This phase was undertaken for a 6-month period, during which all participants received their daily dosage of methadone under the direct supervision of a trained health staff member. This marked the beginning of the outcome testing of the TC intervention.

After 6 months, the participants completed: (1) random urine analysis for opium/heroin residuals via thin-layer chromatography (TLC) to measure drug use, (2) the follow-up study questionnaire (to measure practice, knowledge, and attitude change), and (3) HIV, HBV, HCV viral infection testing. The study also issued and collected self-report behavior cards at four equal intervals over the 6 month period (at the end of months 2, 3, 4 and 5). The study benefited from applying these cards in two ways: to remind participants of the high risk for contracting viral infections (i.e., HIV, HBV, HCV) with risky behaviors (e.g., sharing sharp instruments such as a razor, tattooing, injecting drugs, sexual contact without using a condom), and to collect self-reported engagement in risky behaviours.

## Results

### Participants

Of the 150 participants enrolled in the study, all reported to be of Iranian and Muslim descent, with 62% born in the province of Tehran. The mean age was 31.4 years (SD = 8.2). Only 21% graduated from high school or attended university, while 14% identified themselves as illiterate. Eleven percent of participants reported being unemployed at their time of their incarceration. Fifty percent were single and had never been married, and of those that were married, 65 had children. The mean years of drug abuse was 10.97 (SD = 7.6) and the mean years of injection drug use was 5.39 (SD = 5.21). This translates into participants reporting to have spent, on average, a third of their lives abusing drugs (32% of lifetime, SD = 17.08%), with engagement in injection drug use for approximately one-seventh of their lives (15.98% of lifetime, SD = 12.75%). A total of 103 participants completed the full study.

More than 88% of the participants agreed to be tested for viral infections at the start of the study, of which 42.5% tested positive for HIV, 18.9% for HBV, and 75.9% for HCV. Ninety percent of participants were screened for sexually transmitted infections (gonorrhea and syphilis) and all were negative. A urinalysis was performed on 81% of participants; only one participant tested positive and for a urinary tract infection. Although the findings of this study are unique to Rajaee-Shahr prison and therefore not directly transferable to other TCs in the Iranian correctional system, given the comparable context of HIV, IDU and incarceration across the country, they can provide important insight.

### Stage one

The data collected in the qualitative preparatory stage of the study was systematically reviewed to recommend revisions to the current TC educational and medical interventions for high risk groups, primarily IDUs, in the prison (e.g., cultural adaption). They were originally developed based on the best available knowledge at the time and this study was the first attempt to review the prisoner, staff and prison context for their implementation. Attention was placed during this stage on prisoner behavior change. This stage was time intensive because all interventions needed to be compatible with prison rules and required staff education.

The amended interventions offered through the TC included: counseling and testing for HIV/AIDS and Hepatitis; STI and disease examination (i.e., Venereal Disease Research Laboratory test for syphilis, Fluorescent Treponemal Antibody-Absorption and Gram Staining for Gonorrhea, and Urine Analysis for Urinary Tract Infections), treatment, and care; harm reduction and related drug abuse services, including MMT, syringe exchange, and care and treatment of injection sites; education regarding HIV/AIDS, STIs, and harm reduction; and other related services, such as condom distribution, medication, and treating other diseases such as Tuberculosis. The inclusion of MMT was based on the finding that heroin was the most popular and commonly injected drug among the prison population due to its ease of access, low cost, and efficiency.

### Stage two

Drawing on available data, the outcome indicators include: (1) drug use, (2) change in attitude toward and belief about (knowledge) high risk behaviours, (3) serological conversion (HIV, HCV and HBV), and (4) engagement in risky behaviors (e.g., syringe exchange, condom use).

### Drug use

Throughout the TC intervention, random urine analysis using TLC was conducted to detect opium and heroin residuals, a strong indicator of injection drug use. Compliance was limited with only 64% of participants providing samples at the four points. Positive drug results for opium and heroin were high, ranging from 78.9% to 81.6% of participants (see Table 1). The data does indicate a minimal yet consistent decrease over time (from 81.6% to 78.9%), however, this was not significant (p = 0.982).

**Table 1:** Opium and/or heroin use during TC intervention

**Urine test**	**Test result**	**Frequency**	**Percent (of total)**	**Valid percent**
1	Negative	7	4.7	18.4
	Positive	31	20.7	81.6
	Total	38	25.3	100.0
2	Negative	5	3.3	18.5
	Positive	22	14.7	81.5
	Total	27	18.0	100.0
3	Negative	16	10.7	19.5
	Positive	66	44.0	80.5
	Total	82	54.7	100.0
4	Negative	20	13.3	21.1
	Positive	75	50.0	78.9
	Total	95	63.3	100.0

### Practice, knowledge and attitude change

Analysis of the pre and post- self-administered questionnaire data relayed that a change in IDU risky behaviours and associated sexual practices, although modest, was greater in comparison to knowledge and attitude (see Table 2) and also had the least respondent variability among the three components of the HBM. The results of a paired t-test (see Table 3) similarly revealed reduced engagement in risky practices following respondents’ TC attendance (.000). Neither knowledge nor attitude appeared to be affected—positively or negatively.

**Table 2:** Practice, knowledge and attitude change

	**N**		**Mean (% of maximum achievable score)**	**Std. Deviation**	**Percentage of mean score to maximum achievable score**	
	**Valid**	**Missing**			**Minimum**	**Maximum**
Knowledge pre-test	104	46	27.17 (0.69.6)	6.30	0	0.92
Knowledge post-test	53	97	27.63 (0.70.8)	5.29	0.45	0.89
Attitude pre-test	149	1	76.44 (0.73.5)	9.51	0.40	1.01
Attitude post-test	92	58	75.51 (0.72.6)	13.60	0.30	1
Practice pre-test	148	2	11.85 (0.59.2)	3.19	0.05	0.9
Practice post-test	91	59	13.69 (0.68.4)	3.09	0.21	0.95

**Table 3:** Pre and post-test impact of TC on practice, knowledge and attitude

**Variables**	**Sig. (2-tailed)**
Knowledge pre and post-test	.937
Attitude pre and post-test	.687
Behaviour pre and post-test	.000
Combined knowledge, attitude and behavior pre and post-test	.419

Applying multiple linear regression to examine a combined knowledge, attitude and behavior (KAB) score, the only significant determinant was the degree of knowledge, attitude and behaviour pre-test, adjusting for age, employment, addiction history, viral infection status, and education. This may be partially explained by the size of the study population and the loss of follow-up participants. It can, however, be tentatively hypothesized that based on the coefficients a larger sample size may produce statistical significance.

Additional data analyses relayed that post-intervention KAB is significantly related to KAB at baseline (P = 0.05) (see Table 4). Further, individuals with positive test results in HIV (Beta −5.36) and HBV (Beta −4.48) and also older in age (Beta −0.16 per 1 year increase in age) had acquired scores in KAB, although it was not statistically significant. Age may have an inverse effect on the KAB score as may viral infections (HIV and HBV). Both education and employment may have a positive effect.

**Table 4:** Knowledge, attitude and behaviour post-test association with independent factors

	**Beta coefficient**	**P value**
KAB pretest	0.31	0.052
Age	−0.16	0.719
Employment	4.48	0.76
Addiction history	0.27	0.521
HIV + infection	−5.36	0.383
HBV + infection	−4.48	0.483
Education	1.45	0.75

### HIV, HBV and HCV conversion

**Figure 1:**
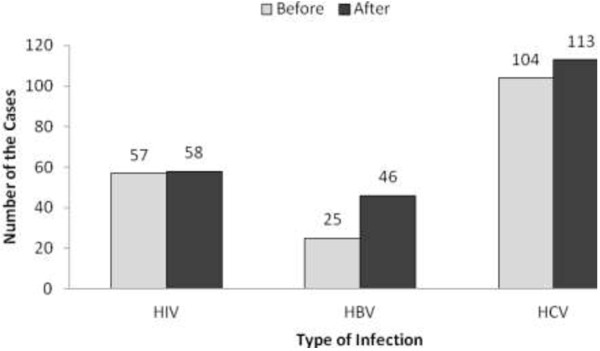
Viral infection status of study participants before and after TC intervention.

The findings of the questionnaire relaying a slight reduction in IDU-related risky behaviour and this is consistent with the *viral infection test results*, which showed an increase in 1 HIV conversion, 21 HBV, and 9 HCV (see Figure 1). Although comparison data on conversions prior to the TC are not available, these findings are encouraging among a high risk group of IDUs. Recall that at baseline 43% of respondents tested positive for HIV, 19% HBV and 76% HCV.

### Risky practices

The completion rate for the *self-report behaviour cards* documenting the participants’ high risk drug and sexual-related practices during the TC intervention was limited (see Table 5). Nonetheless, the data relay that over the course of the 6 month study, 53% of the study participants remained free of any risky behaviour after two months of involvement in the intervention. Engagement in risky behavior increased throughout the 6 month period, with only 1.4% of the participants reporting being risk-free after the total period of the study (i.e., 6 months) (see Table 6).

**Table 5:** Frequency of high risk practices on self-report behaviour cards

	**Returned**	Missing*	**Total**
Time 1	72	78	150
Time 2	57	93	150
Time 3	41	109	150
Time 4	68	82	150

*Includes individuals who did not complete the study.

**Table 6:** Timing of prisoners’ practicing of risky behaviour

**Interval**	**End of month**	**Total**	**Engaged in risky behavior**	**Lost**	**Survival**	**Std. error**	**[95% Conf. Int.]**	
1	2	110	52	0	0.527	0.047	0.43	0.615
2	3	58	14	0	0.40	0.046	0.308	0.489
3	4	44	31	0	0.118	0.030	0.066	0.186
4	5	13	9	4	0.014	0.014	0.004	0.067

More specifically, focusing on HCV converted status because it had the highest initial viral infection rate among the prison population and therefore the greatest availability and accuracy of immediate test results, when risky practices at any of the four time points during the 6 month TC intervention were tested among participants who became HCV positive, the data collected on the self-report behavior cards showed that there was no significant difference between the risky behavior of converted cases and others (fisher’s exact test: 2sided = 1.00) (see Table 7).

**Table 7:** Practicing of risky behaviors among participants who became HCV positive during the study

		**Converted HCV test**		**Total**
		No	Yes	
Risky behavior	Yes	90	5	95
	No	0	0	0
	Missing	34	1	35
Total		124	6	130

Although not identified in the initial study design, two additional indicators of high risk behavior emerged as the study progressed. These were the frequency of abscess formation at the injection site, and the number of drug-related quarrels. According to informal clinical observations and security records, there was no evidence of either of these issues among the study participants. This again suggests that there was some reduction in risky behaviors.

### Risky practices index

**Figure 2:**
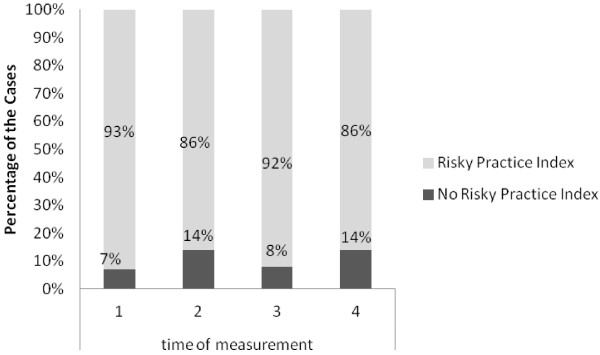
Proportion of participants engaging in risky practices index over the 6 month TC intervention.

In acknowledgement of the cultural taboo associated with intoxicant use in Iran and its potential impact on full disclosure in the study, an index for participating in risky practices related to IDU was created through a combining of reported shared drug injection equipment and positive urine analysis. The findings relayed that 71% of participants engaged in at least one high risk behaviour during the TC intervention, despite receiving the TC harm reduction services. With 29% of the sample missing, it is unknown if any participants were totally free from risky behavior during the 6 month study period. Overall, participation in risky behaviour varied between 93% and 86% at the four reporting points over the course of the TC intervention (see Figure 2).

## Discussion

The documented number of HIV cases in Iran increased 87% between 2001 and 2007 [23]. The vast majority of new cases were identified among IDUs [24-26]. The two key risk factors are imprisonment and the use of contaminated injection equipment while incarcerated [9]. Likewise, recent studies of HCV among men incarcerated in Tehran, Iran identified a history of incarceration to be independently associated with HCV infection [27]. The same was found of HBV and sexually transmitted diseases [27,45]. The same cannot necessarily be applied to female prisoners in Iran [28]. A recent study of TCs in Hamadan province in Iran concluded that the establishment of TCs is a starting point for organizing IDUs infected with HIV/AIDS to detect and address their disease [29]. It is well established that there is a high prevalence of high risk behaviors among IDUs [2], but there remains limited understanding about how to change the high risk behaviours of incarcerated individuals who inject drugs, including the immediate and long-term outcomes of TCs.

Although time consuming, it was critical that the first step in this study focused on generating knowledge about the prisoner population so that the existing Rajaee-Shahr TC educational and medical interventions could be appropriately modified. For example, it was established that the low literacy level among the majority of prisoners who would access the TC needed to be considered in the available written material. Offering services at the TC that are tailored to respond foremost to client needs is foundational to a harm reduction philosophy. It was also essential that baseline characteristics of the prisoner population were established to undertake step two of the study and the identification of outcome indicators, such as HIV, HCV and HBV status, which have been identified as particularly problematic among individuals who inject drugs in Tehran, Iran [30].

The findings of this study relay that the immediate outcome of the TC in Rajaee-Shahr prison on behaviour change among injection drug users is modest. The premise of the Health Belief Model – that individuals’ attitudes and beliefs explain and predict health behaviors – did not support behaviour change over the six-month period. This does not mean, however, that the TC is ineffective. In fact, evidence to the contrary was concluded. It should also be kept in mind that Rajaee-Shahr prison is an institution in which individuals are sentenced for serious, long-term crimes and the volunteer sample for this study represents long-term, high risk IDUs. Consequently, the impact of harm reduction measures on knowledge and attitude may be minimal in the sort-term, even though some behavior was modified. A study by Shams et al. (2011) examined the impact of harm reduction approaches in Iranian prisons over a two year period and found there was not an immediate reduction (after one year/2007) in social behavior across the provincial institutions (e.g., self-harm, drug abuse, bullying), but that over the long-term (2 years/2008) there was [20].

Examining this study’s outcome indicators, it was concluded that introducing the TC intervention led to a minimal decrease in drug use (i.e., opium and heroin), although it did consistently decrease over the 6 month period. Once again, the high risk IDU prisoner population needs to be considered in interpreting this finding. This is a particularly important finding given that all participants in the study were prescribed MMT. Reasons for the participants’ continued drug use while on MMT requires exploration, including whether it is in the individual’s best interest to be prescribed methadone. Future studies should investigate MMT dosage guidelines, means of distribution to combat diversion, and the source of problematic drug use within the prison environment in order to provide the most effective TC harm reduction intervention.

Although the majority of participants continued their drug use, there was still some reported decrease in associated risky practices, although this was not sustained by the vast majority of participants over the six month intervention. With participants reporting long-term drug use, including IDU, it should not be expected that a swift change in behavior would occur. Although, a recent study of prisoners in Ghezel Hesar prison in Tehran, Iran found that “the rate of injecting in the prison unit … decreased drastically since introducing the MMT program” [31]. Further examination into the specifics of this intervention would be useful.

The corresponding absence of change in knowledge and attitude in the current study may in part be explained by the primary focus of the TC being on behaviour change, and the fact that individuals enrolled in the study may have already had altered beliefs and attitudes based on the preparatory stage of the study/at baseline. This finding may also suggest that access to harm reduction services, including MMT, has the potential to motivate prisoners to change their behavior without altering their current knowledge or attitude. This is an important consideration to follow-up on, given that long-term change in high risk behaviors, according to the Health Belief Model, requires change among all three components. This may in fact help to explain the lack of sustained behaviour change across the 6 month study period.

There was no difference found in engagement in risky behaviour among participants who became HCV positive during the study and those that did not. This implies that a ‘one size fits all’ approach to practice change may not be warranted, which underpins the harm reduction philosophy of a TC. There is a need for much greater understanding about the intricacies of prisoners’ lives and their high risk drug use and sexual practice related behaviors [32].

It was also found that HIV and HBV positive status as well as older age may diminish change in knowledge, attitude and practices. This may be explained by the feeling of hopelessness that may accompany a positive viral status and pessimism with older age. This is supported by the finding that the effect was stronger for HIV infection. The opposite may be the case for participants with higher education and employment status. A 2010 survey of injection drug users in Iran similarly found that education greater than high school and permanent employment were protective factors for HIV transmission [6].

The results of the current study suggest that existing interventions may not be as effective for extremely high risk groups like drug injecting prisoners who already are affected with viral infections and are well informed given their compromised health status; hence this may indicate the need for targeted interventions for specific groups [33].

It is well established that not enough is known about the high risk behaviors of IDUs in Iran, both generally and particularly within prison sub-populations [31,34]. A baseline study of prisoners in Karaj Central prison, for example, found that injecting, in comparison to non-injecting drug-using prisoners, were more likely to have been tested for HIV infection [10]; this is useful information. In measuring the outcome of the TC, the current study only considered injection drug use and related risky practices. To further develop the TC, it would be useful to know more context-specific information, such as if the TC stimulates change in the extent to which drug users organize their lives around drug use, how much drug use is integrated into their lives, and the ways in which drug use negatively impacts other aspects of their health [35]. Related, it was determined that the most frequent viral infection among the participants was Hepatitis C. This is consistent with other studies of incarcerated groups [33], and should be given equivalent attention to HIV in the reduction measures being implemented within TCs.

Given the complex nature of this study, it has four key limitations. These may in part explain the absence of concrete findings. First, the attempt to measure both sex and non-sex related high risk behaviors in the conservative social environment of a prison setting in Iran has extreme cultural and religious taboos. This may have translated into decreased levels of accuracy and compliance with reporting in the study. One means to address this was the development of a risky practices index (combined indicator of reported shared drug injection equipment and positive urine analysis results at the same sequence). In the future, the development of a less complex and lengthy questionnaire may want to be considered, as well as observational reporting. Related, focus was paid to measuring prisoner behavior to the exclusion of considering the mutual role of staff in the TC and the prison environment (other than in the preparatory phase) in this study. Third, participant retention was a limitation of the study; it is anticipated that with an increased, non-punitive presence of the TC clinic within the prison environment as time progresses, prisoners may be willing to report their IDU and associated risky practices. And fourth, the use of thin-layer chromatography as the form of urine analysis in this study has potential problems in that it may produce incorrect conclusions; it can produce both a false positive and a false negative for various reasons, including the presence of drug metabolite-like chemicals in urine and human error. Although more expensive, more accurate tests may want to be applied in future studies.

Although the findings of this study are specific to Rajaee-Shahar prison in Tehran, Iran, it remains that the Iranian prisoner “population is under-served by HIV prevention services, as are the prison populations of many countries in the Middle East and Eastern Mediterranean regions” [12]. This recognition is transferable to other countries in the world. As the Iranian working group on drug treatment and rehabilitation in prison settings shared: “We believe that Iran with the unfortunately large addiction affliction has much to offer and in return is eager to learn when it comes to clinical interventions in drug abuse” [15]. Thus this study makes a contribution to both understanding harm reduction practices within an Iranian prison and the international literature. As correspondent Ian Tanner writes for *The Majalla*, an Arab magazine, “Despite the draconian policy towards punishing trafficking and the fact that treatment is not universally available, Iran’s drug policy is at the very least surprising, and perhaps even inspiring” [11].

## Conclusion

The results of this study relay that the TC in Rajaee-Shahr prison had modest immediate impact (i.e., 6 months) in reducing high risk harmful behaviors among incarcerated IDUs. In its current form, it is unknown if the TC would bring about a sustainable, long-term reduction in harmful behaviors. This is particularly important considering the lack of significant change in knowledge and attitude related to high risk practices; the three together comprise the Health Belief Model. Also, considering the large number of missed cases at follow-up, it may further indicate the clinic’s benefits were either not evident or relevant to the participants. Past studies have shown that harm reduction initiatives are most effective when perceived benefit is high among participants [27]. Future research is required to determine the specific gaps in the current TC model.

In order to combat the HIV/AIDS epidemic in Iran, the results of this research must be considered not only in the context of the prison environment, but beyond to the general Iranian population [26]. The majority of the prisoners in Rajaee-Shahr prison will be released and will reintegrate into the general population where they have the potential to spread HIV and other viral infections [20]. We know from other studies that injection drug users engage in high risk behaviors outside the prison environment [2]. We also know that there is a need for practice and research-focused attention to be placed on the transition from prison to the community, and keeping up safe practices such as MMT. A recent study of HIV/AIDS in Iran emphasized “the need for intensified HIV prevention efforts with men who use drugs via injection and strengthened efforts to encourage the individual at risk to get tested for HIV” [26]. With a large number of IDUs filtering through the prison system in Iran, and the high rates of HIV, HBV and HCV, this may be an ideal catchment area. This is supported in other studies [36,37]; in the general Iranian community, people who wish to determine their HIV status are oftentimes prevented from doing so because of the sociocultural context. As a result, the impacts of the triangular clinic examined in this study should be supported as one part of a broad, multi-faceted HIV/AIDS and other health behaviour prevention strategy in the Islamic Republic of Iran.

## Competing interests

The authors declare that they have no competing interests.

## Authors’ contributions

RTA and BE participated in the design of the study, collection of data, analysis and interpretation of data, drafting the article, and final approval of this version. CAD and KT participated in contextualizing the data, drafting the article, and final approval of this version. PA, MH and AM participated in reviewing drafts of this paper and final approval of this version. All authors read and approved the final manuscript.
